# Cultural ecosystem services provided by the Baltic Sea marine environment

**DOI:** 10.1007/s13280-019-01239-1

**Published:** 2019-08-31

**Authors:** Heini Ahtiainen, Eero Liski, Eija Pouta, Katriina Soini, Christine Bertram, Katrin Rehdanz, Kristine Pakalniete, Jürgen Meyerhof

**Affiliations:** 1 Natural Resources Institute Finland, Latokartanonkaari 9, 00790 Helsinki, Finland; 2 Kiel Institute for the World Economy, Kiellinie 66, 24105 Kiel, Germany; 3 University of Kiel, Kiel, Germany; 4 AKTiiVS Ltd, Riga, Latvia; 5 Institute of Landscape Architecture and Environmental Planning, Technische Universität Berlin, Strasse des 17. Juni 145, 10623 Berlin, Germany; 6 Department of Economics, Kiel University, Olshausenstrasse 40, 24098 Kiel, Germany

**Keywords:** Baltic Sea, Compositional data, Cultural ecosystem services, Marine environment, Preferences

## Abstract

This paper applies the concept of cultural ecosystem services (CES) to reveal the diverse benefits the Baltic Sea provides to human well-being. The study identifies and defines relevant CES for marine and coastal environments and applies them in a survey with 4800 respondents from Germany, Finland and Latvia. The relative importance of various CES was determined by asking respondents to allocate 100 points between CES related to recreation, landscape, inspiration, learning and education, spiritual experiences and belonging, historically and culturally important places and the existence of habitats. The results reveal significant differences in the importance of various CES across countries, users and nonusers of the Baltic Sea, as well as respondents with different human–nature relationships. The results emphasize the importance of considering recreation, landscapes and habitats in conservation policies, while acknowledging that all CES are perceived as important by some population groups.

**Electronic supplementary material:**

The online version of this article (10.1007/s13280-019-01239-1) contains supplementary material, which is available to authorized users.

## Introduction

The cultural ecosystem services (CES) concept relates to services provided by ecosystems in terms of their life-enriching and life-affirming contributions to human well-being (Fish et al. 2016). In contrast to provisioning and regulating services, CES “cover all the non-material, and normally non-extractive, outputs of ecosystems that affect physical and mental states of people” (Haines-Young and Potschin 2012). CES include spiritual enrichment, cognitive development, reflection, recreation, aspects related to the existence of a healthy ecosystem and aesthetic experiences (e.g. MEA 2005). As observed by many authors, little attention has been paid to CES compared to the other ecosystem services (ES), especially for the marine environment (Baulcomb et al. 2015; Martin et al. 2016; Rodrigues et al. 2017). Since 40% of the world’s population live within 100 km of the coast (UN ATLAS OF THE OCEANS 2002–2016) and CES form from the interaction of people and nature, we can assume CES from the coastal zone and marine areas close to coastal populations have a high importance (Brown and Houser 2017; Rodrigues et al. 2017). These areas are also typically under strong pressure from pollution originating on land, and heavy use of marine areas. This trade-off implies that information about CES have high relevance for management decisions. In this paper we focus on the whole range of CES provided by the Baltic Sea in order to produce information about their importance to the well-being of people around the Baltic Sea. Making the whole range of CES visible enables their consideration in marine policies and management of the Baltic Sea.

Previous studies of CES reveal two central knowledge gaps. First, studies have a tendency to focus only on specific services, typically recreation and landscape aesthetics (Hernández-Morcillo et al. 2013; Liquete et al. 2013; Milcu et al. 2013). Also for the marine environment, research has focused on only a few CES [for recent reviews of the literature see Martin et al. (2016) and Rodrigues et al. (2017)]. In these studies, recreation is the most frequently addressed service, followed by cultural heritage and aesthetic values, educational values and spiritual and religious values. Social relations and knowledge systems are the least addressed services. The lack of quantitative or qualitative assessments of all CES partly originates from the lack of suitable methodologies and indicators to assess them simultaneously (Rodrigues et al. 2017). Milcu et al. (2013) argued that the overemphasis on recreation and tourism highlights the general difficulty in measuring other, often less concrete, CES. The difficulty in measuring some CES also leads to the issue of reappraising the feasibility and applicability of the existing CES classifications. Especially in case of coastal and marine environments, whether some CES overlap, or if useless classes are present in the existing classifications, requires further assessment.

Secondly, even if the whole range of CES were quantifiable, including all of the services from a given framework [such as MEA (2005), TEEB (2010) or CICES (see Haines-Young and Potschin (2012)], there is still a lack of understanding regarding what explains peoples’ perceptions of the importance of CES categories. Few, if any studies have explicitly pointed out how individuals’ characteristics relate to perceived importance of CES. The relative abundance of coastal and marine CES depends on the characteristics of the coastal ecosystem of a country (Brown and Houser 2017), but there are also social and economic differences among countries. For the Baltic Sea, Ahtiainen et al. (2013) and Czajkowski et al. (2015) compared the use of recreational services among nine coastal countries. In these countries, the proportion of respondents who had visited the Baltic Sea at least once ranged from 49% in Russia to over 97% in Sweden, and the number of trips from 0.5 per year in Russia to 6.4 in Sweden. Differences have also been found in the existence values related to healthy marine ecosystems in cross-country comparisons of the Baltic Sea (Kosenius and Ollikainen 2015). For example, Swedes favoured a large improvement in fish stocks over a large improvement in pristine areas, contrary to Finns. Sagebiel et al.’s (2016) review of studies on monetary valuation of Baltic Sea ES indicates that recreation is emphasized most among valuation studies of CES. Nieminen et al. (2018) present descriptive information on the importance of CES in Finland, but do not analyse differences between individuals. These previous studies reveal that the importance of CES can vary among countries and among individuals within a country, though knowledge about the factors explaining the relative importance of individual services in the whole range of CES is missing.

To fill these knowledge gaps we (1) develop a measure that covers the entire CES range. Using this measure in the three geographically, socio-economically and culturally different Baltic Sea countries Finland, Germany and Latvia, (2) we identify the relative importance of various CES for the Baltic Sea region. Then, (3) we identify the determinants that explain the individually-perceived importance of different CES using compositional statistical analysis. The determinants we focus on include sociodemographic, attitudinal, geographic and recreation-related factors. We further group individuals based on their perceptions of CES. We (4) identify categories of CES that are perceived as separate and those that cluster with each other, in order to evaluate the feasibility of CES classification. Finally, (5) we discuss the implications of the relative importance of CES for the management of the Baltic Sea.

## Factors underlying perceptions of CES from marine environments

In developing a CES measure we take into account critical discussions concerning the CES concept. CES often occur in bundles (Raudsepp-Hearne et al. 2010) and people cannot clearly separate the benefits provided by one CES subcategory from another. Moreover, intangible CES, such as spiritual and inspirational services, are not usually associated with specific landscapes or sea-scapes, as are recreational services. There are also synergies in CES that complicate identification of each service’s importance. For example, recreation might be valuable due to aesthetic qualities or the cultural heritage of a site, and likewise, areas with scenic beauty often provide inspiration and opportunities for education. There might also be synergies between cultural heritage and identity, and the creation of social relations that occur together, for example, in the case of recreational fishing (Fletcher et al. 2014). The fuzziness of relationships among the services may risk double counting (Gould et al. 2014). Furthermore, the difficulties in measuring the whole range of CES may lead researchers and policymakers to assume that, for instance, recreation and landscape values represent CES to the greatest extent. Omitting CES other than recreation and tourism may also lead to their unconscious marginalization (Milcu et al. 2013). These critical standpoints support a measure of CES that leads individuals to express the relative importance they place on various CES categories.

In explaining the relative importance of CES categories for people we acknowledge the contextual nature of CES, that is, they require an interpretative perspective or “lens” rooted in one’s cultural background (Baulcomb et al. 2015), held values (Van Riper and Kyle 2014) and an understanding of the “place” under exploration (Flint et al. 2013) and consequently highlighting the plurality among individuals (Cooper et al. 2016; Kenter 2016). With this multiformity associated with CES, the concept extends the understanding of human–nature relationships and the ways in which people relate to and behave in the environment, and in particular of their preferences for environmental change (Flint et al. 2013; Baulcomb et al. 2015). CES also emphasize the role of aesthetic and spiritual understandings of nature’s value, which are not only considered a benefit, but may also lead to moral responsibilities towards nature (Cooper et al. 2016). Moreover, as Pleasant et al. (2014) note, CES are the only ES category that can be linked to all four types of human well-being: security, health, good social relations and the basic materials for a good life. In principle, CES provide an approach for extending the understanding of the human–nature relationship (Fish et al. 2016).

In this paper, we focus on the association between individuals’ human–nature relationships measured with the New Environmental Paradigm (NEP) (Dunlap et al. 2000) and their perceptions of the various classes of CES. The NEP scale measures nature-based, ‘primitive’ beliefs that reflect an individual’s fundamental priorities concerning human–nature interactions (Oskamp and Schultz 2005). The NEP has already been used in a few studies to explain individuals’ perceived ES. Van Riper and Kyle (2014) conducted a study on Santa Cruz Island and found that people with strong biocentric attitudes or world views, that is, located on one end of the NEP scale, particularly emphasized the scientific and biodiversity value of the environment, while people associated with neutral measures on the NEP scale emphasized recreational values. Moreover, the groups with strong biocentric attitudes assigned their values to national parks and nature protection areas without necessarily accessing them. While Van Riper and Kyle (2014) focused on recreationists’ perceived values from ecosystems and human-nature relationships, in our case we are also interested in value perceptions that are not related to actual use of the site.

In order to explore and explain the differences in perceptions of CES, we acknowledge the ideas of the relational approach to CES. The relational approach suggests that people actively create CES through their interactions with ecosystems, rather than considering them as products of nature that people utilize for a particular benefit to well-being. The services are understood as a combination of environmental spaces, cultural practices (such as playing and exercising, creating and expressing, producing and caring, gathering and consuming) and the benefits that result from the processes and entities that people actively create and express through interactions with ecosystems and that affect their identities, experiences and capabilities (Fish et al. 2016). The practices are not necessarily only physical or embodied, but also textual and mediated or linguistically discursive in their form. For example, benefits may arise from reading and watching magazines, cinema, art and literature that carry motifs and narratives of the ecosystems in question without a person ever visiting the natural asset. This elaboration and extension to practices allows us to understand that people may value some CES categories even though they are not users of these services in the conventional or physical sense. Our analysis identifies the CES categories that are especially use and non-use related.

Taking into consideration these prerequisites we developed a novel measure that tackles the recognized challenges to better identify the different CES and their relationships, and tested it in the Baltic Sea region with geographically and culturally varied coastal countries.

## Methods

### Measuring the importance of CES

To develop the measure for CES, we applied the Common International Classification of Ecosystem Services (CICES) developed from the work on environmental accounting undertaken by the European Environment Agency (Version 4.3; Haines-Young and Potschin 2012). CICES aims to provide a comprehensive classification by introducing the idea of a five-stage hierarchical structure to describe and measure ecosystem services: sections, divisions, groups, classes and class types. To account for context, place and culture, we needed to operationalize an ES classification for the Baltic Sea context. We used the 11 ES classes for CES from CICES as a starting point (see column two in Table 1). From these classes we developed a more detailed specification of the ES classes for the Baltic Sea ecosystem. These Baltic Sea examples are presented in column three of Table 1.

**Table 1:** CICES-based description of cultural ecosystem services provided by the Baltic Sea

CICES group	CICES class	Applications to the Baltic Sea	Specification of CES used in the survey	Abbreviation
Physical and experiential interactions with biota, ecosystems, land-/sea-scapes [environmental settings]	Experiential use of plants, animals, land-/sea-scapes in different environmental settings	Bird watching, diving, snorkelling	Opportunities for recreational activities (e.g. swimming, angling, walking, boating, bird watching)	Recreation
	Physical use of land-/sea-scapes in different environmental settings	Being on the beach, swimming, walking, hiking, boating, angling, hunting, bird watching, photography		
Intellectual and representative interactions with biota, ecosystems, land-/sea-scapes [environmental settings]	Scientific	Ecological, social and cultural research on the Baltic Sea environment	An environment for learning and gaining new information	Education
	Educational	Environmental education, e.g. literature, lessons, camps, excursions		
	Heritage, cultural	Literature on the culture of the Baltic Sea, museums, ruins, cultural landscape	Experiencing historically and culturally important places	Historic
	Entertainment	TV programmes, multimedia, literature on the Baltic Sea	Not included as such	
	Aesthetic	Paintings, music, performances inspired by the Baltic Sea ecosystems	Inspiration for artistic work (photography,…)	Inspiration
			Enjoyment of landscapes	Landscape
Spiritual and/or emblematic interactions with biota, ecosystems, land-/sea-scapes [environmental settings]	Symbolic	Charismatic species (seals, fishes, birds) or other objects that represent, stand for or suggest an idea linked to the Baltic Sea environment	Spiritual experiences, sense of belonging, symbolic meaning	Spiritual
	Sacred and/or religious	Spiritual, ritual identity, holy places, sacred plants and animals and their parts		
Other cultural interactions with biota, ecosystems, land-/sea-scapes [environmental settings]	Existence	Enjoy knowing that the Baltic Sea exists	Habitats for many animals and plants	Habitat
	Bequest	Future generations able to enjoy the Baltic Sea environment	Not included as such	

To deduce the relative importance of CES we designed a question in which respondents had to distribute 100 points according to the importance they attach to the various CES provided by the Baltic Sea. Using such a relative measure allows us to deduce the importance of a particular CES relative to other CES. However, to apply such a question, the number of CES categories had to be kept reasonably low, the categories had to be sufficiently distinct from one another and they had to be phrased such that they were easily understandable for lay people. For this reason, the ES used in the survey were reduced to seven categories following Kandziora et al. (2013). Moreover, the wording was shortened and kept as simple and understandable as possible (column three in Table 1).

This operationalization of CES was tested with Latvian and Finnish focus groups of citizens. The groups discussed the reasons for which they value the Baltic Sea based on the presented CES. In addition, the method for distributing points was tested and found to work reasonably well, even though adding up the points was found slightly difficult with pen and paper. In the final internet version of the survey, points were added up automatically so that respondents did not have to carry out any calculations themselves. The seven categories as described in the last column of Table 1, plus an option for “other reasons not mentioned in the list”, were included in the pilot survey and in the final survey. For full phrasing of the survey measure, including the question, see Appendix S1.

To test the assumption that perceived CES from the Baltic Sea region are shaped by the more general human–nature relationship of a respondent (DeGroot and Steg 2008), we measured respondents’ ecological attitudes using the NEP (Dunlap et al. 2000). The NEP measure with a five-point scale (from totally agreed to totally disagree), encompasses statements with the following facets: (1) balance of nature, (2) limits to growth, (3) risk of an eco-crisis, (4) anthropocentrism and (5) humans’ ability to control nature. The final NEP measure is produced from the sum of these statements.

Information regarding recreational use of the Baltic Sea during the previous 3 years made it possible to separate nonusers from users. Mapping the home location of the respondents allowed us to calculate the distance from home to the Baltic Sea, and also to define the urbanization level of the home location. In follow-up questions respondents provided information on gender, age, education, income, occupation and household size.

### Survey implementation

To develop the CES measure and to test it in the Baltic Sea context, new survey data were collected in Finland, Germany and Latvia to reveal the diverse benefits to human well-being from the Baltic Sea. The survey was designed with international cooperation in 2015–2016. Pre-testing included expert reviews by researchers in environmental valuation and marine ecology, focus groups (one in each country) and a pilot survey in each country in June–July 2016.

The final survey was implemented between November 2016 and February 2017. It was targeted at residents of each country using stratified random sampling that was representative of the population in each country. Stratifying, for instance, according to age, gender, location and educational level ensured representative samples of the national populations. For Germany, coastal regions were oversampled to increase the share of Baltic Sea visitors in the final sample. The primary data collection method used in Finland and Germany was computer-assisted web interviews (CAWI) with internet panels (Table 2). The implementation method in Latvia combined computer-assisted personal interviews (CAPI) and CAWI to ensure a representative sample of respondents in all age groups (including older age groups for whom internet use is insufficient in Latvia to achieve the representativeness by CAWI) was obtained. The CAPI were conducted at the respondent’s place of residence. Altogether, 4,800 respondents answered the survey, with a little over 2000 respondents in Finland and in Germany, and around 760 in Latvia. The average response time was around 20 min.

**Table 2:** Survey implementation

Country	Finland	Germany	Latvia
Survey mode	CAWI	CAWI	CAWI and CAPI
Sample size (completed responses)	2048	2005	759 (CAWI: 351, CAPI: 408)
Response rate (%)	34	15^a^–20^b^	26.7 (CAWI: 18.5, CAPI: 43.3)
Age of sampled individuals (years)	18–79	18–77	18–74
Survey company	Kantar TNS (formerly TNS Gallup)	Lightspeed Research GmbH	Latvijas Fakti Ltd.

*CAWI* computer-assisted web interviews with internet panels, *CAPI* computer-assisted personal interviews

^a^Nationally representative part of the survey with random sampling stratified by age, gender and state (800 respondents)

^b^Sample drawn only from coastal areas (1200 respondents)

Table 3 presents descriptive statistics for the final data and the corresponding national statistics. There were slightly more men in the German and Latvian data sets compared to the national population. In all three data sets, average age was somewhat higher than the average of the populations, while the share of respondents with higher education was slightly lower.

**Table 3:** Descriptive statistics for survey respondents, and corresponding national statistics

Variable	Finland		Germany		Latvia	
	Sample mean (S.D.)	Population mean	Sample mean (S.D.)	Population mean	Sample mean (S.D.)	Population mean
Age in years	46.8 (17.3)	42.5	48.6 (11.6)	44.3	45.2 (15.6)	42.1
Male	0.46 (0.50)	0.49	0.54 (0.50)	0.49	0.48 (0.50)	0.46
Higher education	0.37 (0.48)	0.44	0.27 (0.45)	0.28	0.24 (0.42)	0.24

Sources of population statistics: statistics Finland 2016, CSB Gov 2016, Destatis 2015

The survey included 41 questions and 6 sections. After an introduction to the survey and the Baltic Sea, respondents’ recreation visits were mapped and visit information collected. Reponses towards changing environmental conditions were collected. The importance of CES from the Baltic Sea was measured before the set of background questions.

### Statistical methods

The dependent variable in the statistical analysis was the whole CES measure consisting of eight elements based on survey responses to the importance of CES, named as: recreation, landscape, inspiration, education, spiritual, historic, habitat and other services. These were denoted R1, R2, R3, R4, R5, R6, R7 and R8, respectively. Any vector *x* with non-negative elements *x*_1_, …, *x*_*D*_ representing percentages of the total is subject to the obvious constraint $x_{1} + \cdots + x_{D} = 100$. As these individual services were subject to this constraint, and only have importance in relation to each other, we have a compositional dependent variable. Compositional Data Analysis is the standard statistical method used when data contain information about the relative importance of parts of a whole, typically with a fixed sum (Aitchison 1986). In the statistical modelling, the aim was to explain the differences in the compositions, that is, the relative importance of the CES between individuals. Compositional data pose certain restrictions with respect to traditional statistical analyses, and thus special treatment is required (Aitchison 1986). We performed an isometric log-ratio (ilr) transformation on the dependent variable and performed compositional data analysis using the ilr-transformed variable as the dependent variable [see Egozcue et al. (2003) and Ahtiainen et al. (2015) for more details]. We fitted several models with predictors describing the respondents’ sociodemographic characteristics (e.g. gender, age, household size, occupation, income, education), geography (e.g. country, distance to coast, category of place of residence), recreation (user or nonuser) and human–nature relationship (NEP scale) (see Table 4).

**Table 4:** Explanatory variables used in the models

Variable	Description
distcoast	Distance from the respondent’s place of residence to the Baltic Sea coast in km, continuous
caturb	Category of respondent’s place of residence, 1 = urban (share of rural population < 20%), 2 = intermediate (share of rural population 20–50%, 3 = rural (share of rural population > 50%)
nonuser	1 if the respondent does not visit the Baltic Sea, 0 otherwise
male	1 if the respondent is male, 0 if female
country	Germany, Finland, Latvia, used as categorical
age	Respondent’s age in years, continuous
sumnep	Sum of the NEP scale variables, including 6 items, continuous
hhsize_under	Number of children under 18 years age in the household, continuous
Income_class	Income class of the respondent, ordinal
low_edu	1 if the education was compulsory school or high school, 0 otherwise
fulltime	1 if the respondent is employed fulltime, 0 otherwise

Zero elements within compositions were assumed to be so called ‘rounded zeros’. We applied the multiplicative replacement strategy presented by Martin-Fernandez et al. (2003), generating imputations for zeros from a continuous uniform distribution unif (0.001, 0.0049).

Due to the compositional nature of the dependent variable, a univariate modelling approach was not meaningful. Thus, we applied a multivariate ANCOVA to examine the joint significance of the influence of each predictor on the composition response. As model selection tools, we used fivefold cross-validation *R*^2^ values and multivariate ANOVA type III *p* values based on an F-distribution approximation of Pillai’s trace. Pillai’s trace takes values in the range of [0,1], where a larger value indicates higher significance of a predictor. We first fitted a model with the main effects for all potential predictor variables, including *distcoast, caturb, nonuser, male, country, age, sumnep, hhsize_under, income_class, low_edu* and *fulltime* (see Table 4 for definitions). In addition, we added interaction terms between *country* and *nonuser*, and between *country* and *sumnep*. To select the final model we dropped each predictor for which the *p* value was greater than 0.05. With the remaining predictors, we found a model that maximizes the cross-validated *R*^2^ value.

Beyond multivariate ANCOVA, we used clustering techniques for the CES composition. Hierarchical cluster analysis, applicable with compositional data, was used to illustrate the clustering of CES with each other. The distance matrix was constructed using Euclidean distance. For clustering with respect to variables, the distance matrix was calculated from the variation matrix. The clustering was performed using the Ward method. We also used the same method to cluster the respondents based on their CES compositions to define and describe the respondent groups that had similarities in the perceived CES.

The statistical analyses were performed with R software (R Core Team 2017) and the package compositions version 1.40-1 (Van den Boogaart et al. 2014).

## Results

### Descriptive results on the importance of CES

Table 5 presents the relative importance of CES based on simple descriptive statistics of the average points allocated to each CES by country and in total. Three CES clearly stand out for their importance: recreation, habitat and landscape. The other CES received, on average, significantly fewer points. Some country-wise differences were also evident. Recreation was important in all countries, especially in Latvia, as were landscapes. Habitats, as a non-use value component, were more important in Germany and Finland than in Latvia. Table 5 also provides the compositional mean of the CES categories and confirms the relative importance of the CES categories.

**Table 5:** Relative importance of different CES in the Baltic Sea (average points out of 100 allocated to each CES) and the compositional mean

Cultural ecosystem service	Germany	Finland	Latvia	Average, all three countries	Compositional mean
Recreation (R1)	26	31	46	31	0.383
Habitat (R7)	24	24	11	22	0.207
Landscape (R2)	23	21	20	22	0.272
Historic (R6)	8	9	5	8	0.055
Education (R4)	5	5	3	5	0.030
Other (R8)	5	4	4	5	0.013
Inspiration (R3)	5	3	5	4	0.023
Spiritual (R5)	3	3	6	3	0.016

### Model results

Table 6 presents the multivariate ANOVA type III *p* values with respect to each predictor from the full model to examine the joint significance of the influence of each predictor on the composition response. We observed that the coefficients were significant at the 5% level with respect to each variable, except for *low_edu* and *fulltime*. Thus, each predictor, except for *low_edu* and *fulltime,* was statistically significant at the 5% level when added last to the model. Model selection was continued by dropping these two predictors.

**Table 6:** ANOVA table of the full model with interactions

Variable	Type III test *p* value
Intercept	0.000
nonuser	0.000
male	0.000
country	0.000
low_edu	0.244
fulltime	0.093
income_class	0.003
caturb	0.001
hhsize_under	0.035
sumnep	0.000
distcoast	0.007
age	0.001
nonuser * country	0.000
sumnep * country	0.000

The cross-validated *R*^2^ value was maximized with the predictors *nonuser, country, sumnep* and *male* with a value of 0.061. The apparent *R*^2^ value of the model consisting of these four predictors was 0.071. This four-predictor model fitted to the whole data set provided statistically significant *p* values for the type III test for each predictor. Thus, we infer that this four-predictor model gives the best overall performance with regard to prediction and interpretability.

Table 7 presents the coefficients of the four-predictor model. The constant of the model was interpreted as the expected composition for the baseline level (*country* = Germany, *nonuser* = 0, *gender* = female, *sumnep* = 0). The parameter corresponding to the male gender, for example, was interpreted as the increment (in the sense of perturbation) on the average response from the baseline (female) to the level ‘male’. In the interpretation, one may compare the estimated composition parameters with the 8-part neutral element (1,…,1)/8. That is, any component with an estimated coefficient greater than 0.125 (1/8) can be seen as having an increasing effect and any component with a coefficient smaller than 0.125 can be seen as having a decreasing effect on the dependent variable, i.e. the importance of the CES subcategory.

**Table 7:** Four-predictor model coefficients

	Recreation	Landscape	Inspiration	Education	Spiritual	Historic	Habitat	Other
Intercept	0.533	0.293	0.045	0.020	0.016	0.035	0.021	0.037
Country = Finland	0.176	0.103	0.084	0.145	0.125	0.148	0.127	0.091
Country = Latvia	0.279	0.077	0.127	0.068	0.260	0.058	0.030	0.101
Nonuser	0.078	0.093	0.118	0.131	0.131	0.120	0.208	0.122
Male	0.154	0.107	0.110	0.110	0.129	0.139	0.094	0.156
sumnep	0.096	0.121	0.101	0.130	0.110	0.128	0.228	0.086

Reference levels: country = Germany, user, female

For Finland, there was an increase with respect to CES related to recreation, education, spiritual, historic and habitat services compared to Germany, meaning that these five CES were more important in Finland than in Germany. Landscape and inspiration were less important. For Latvia, recreation, inspiration and spiritual were more important than in Germany, but landscape, education, historic and habitat less important.

For nonusers, there was an increase with respect to the CES related to education, spiritual and habitat compared to those who had visited the Baltic Sea. Thus, these services appeared more important to nonusers, which seems intuitive since they are not necessarily associated with direct use of the Baltic Sea. Male respondents seemed to have larger values especially for recreation than women and smaller values for habitat. As *sumnep* increased, that is, when a respondent had a more biocentric worldview, the importance of education, historic and habitat services increased, whereas the other categories decreased.

**Fig. 1:**
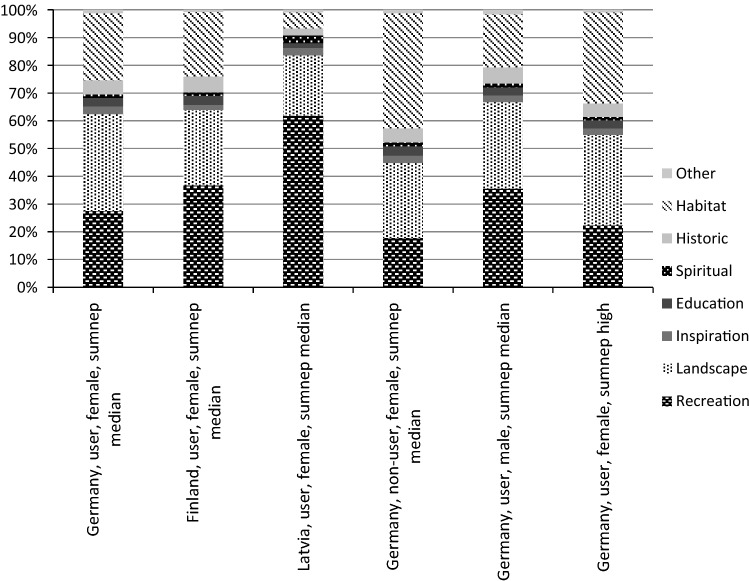
Stacked barplot of predictions of the relative importance of different categories of cultural ecosystem services from the four-predictor model. Abbreviations are as in Table 4

Figure 1 presents examples of predictions for specific predictor combinations. The predictors *country*, *nonuser*, *male* and *sumnep* were of interest. The first bar corresponds to the baseline values of the predictors *country*, *nonuser* and *male* (i.e. Germany, user and female, respectively) and the median value for *sumnep*. Every other bar is compared to this bar while changing the level of one predictor at a time.

The estimated coefficients with respect to each predictor and composition show an increase or a decrease compared to the baseline prediction. For example, the relative weight of recreation was much larger for Latvia than for Germany. Components for inspiration, education, spiritual, historic and other services all had relatively small values. Therefore, we could have combined or dropped these parts of the response composition since they contribute little to the predictions. The effect of *sumnep* was less straightforward. The largest estimated positive effect for *sumnep* was with respect to habitat. We see an increase for habitat when comparing the first and last bars in Fig. 1, which correspond to median and 3^rd^ quartile *sumnep* values, respectively.

### Clustering cultural ecosystem services and respondents

We also performed hierarchical cluster analysis in order to examine the closeness of the response compositions in Euclidean distance, as well as the closeness of individuals.

**Fig. 2:**
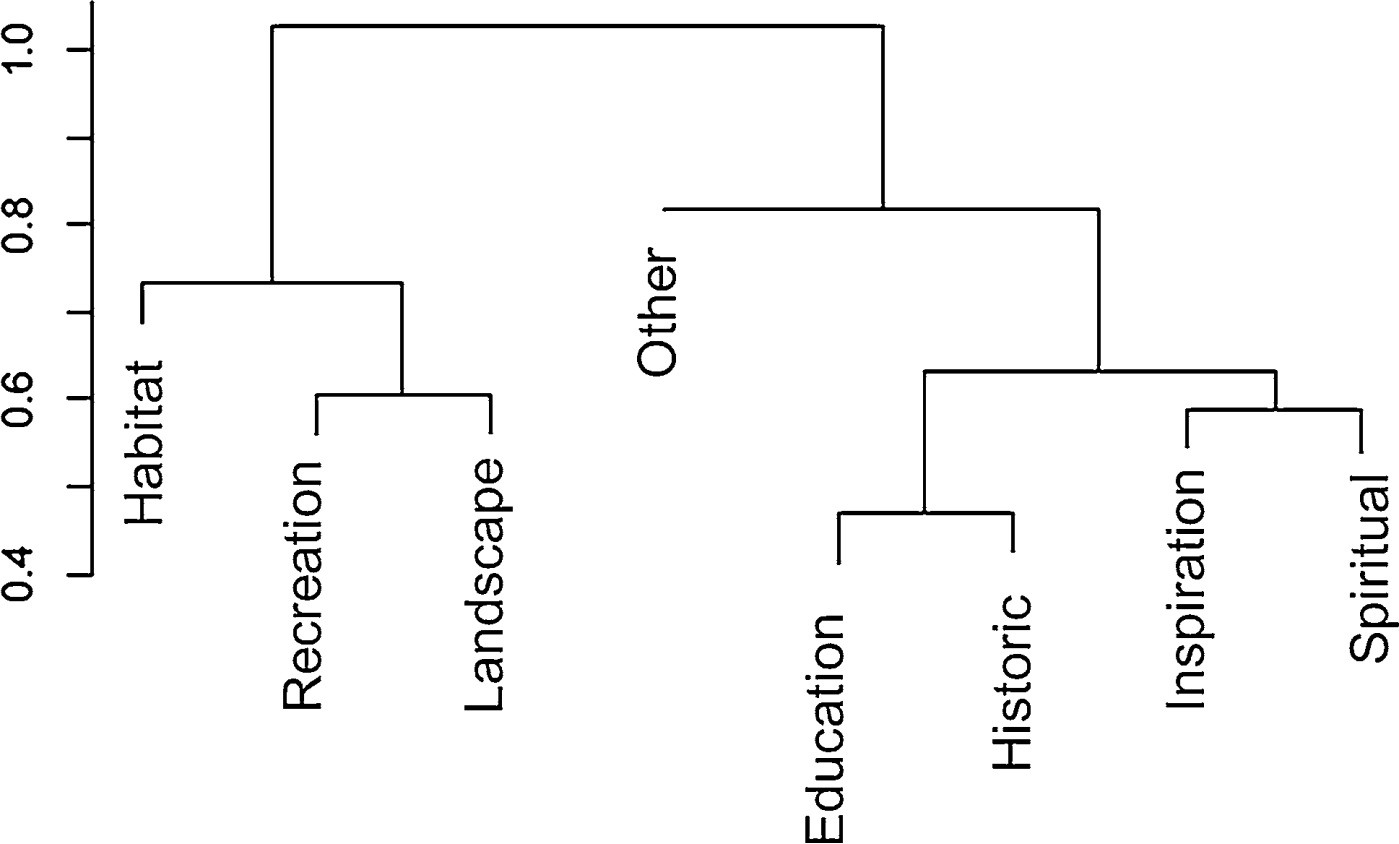
Dendrogram of hierarchical clustering with respect to cultural ecosystem services

Figure 2 presents the dendrogram of compositions, that is, clustering of the different CES based on their closeness. For example, the closeness of inspiration, education, spiritual and historic (R3–R6) components grouped together. These elements each had very small values in the mean composition. Also, the components recreation (R1) and landscape (R2) grouped together. Habitat (R7) grouped last, indicating that it reflects a unique representation separate from the other CES.

Table 8 presents the four-cluster solution of respondents based on the closeness of their perceptions of the importance of different CES. The first cluster (49% of the respondents) considered the existence of habitats for plant and animal species especially important, as well as historically and culturally important places. The members of this cluster were often Finns and Germans, and had the strongest environmental orientations according to their corresponding NEP values. There were fewer recreational users of the Baltic Sea among these respondents. The second cluster (18%) especially emphasized inspiration for art, learning, spiritual experiences and sense of belonging, and experiencing historically and culturally important places, but also the other category of unspecified ‘other’ CES. This cluster had a higher weight among German respondents. The third cluster of respondents (20%) emphasized recreation services, landscape and the existence of habitats for plant and animal species. Membership of this cluster was more common among the Finnish respondents than among the other nationalities, and respondents’ NEP values were higher than average. The fourth cluster (14%) stressed the importance of recreation. Individuals in this cluster were more often users of the Baltic Sea. Latvian respondents were over-represented in this final cluster, which had lower than average NEP values.

**Table 8:** Results of the two-step clustering and the background variable associations among cluster groups (mean values)

	Cluster 1	Cluster 2	Cluster 3	Cluster 4	All		
Size of cluster	1515	534	410	425			
Cultural services compositional mean							*F* test *p* value
Recreation	0.208	0.280	0.399	0.810	0.383	Between clusters	< 0.01
Landscape	0.232	0.177	0.260	0.142	0.272	All cluster comparisons	< 0.01
Inspiration	0.021	0.028	0.007	0.014	0.023		
Education	0.035	0.038	0.009	0.007	0.030		
Spiritual	0.016	0.023	0.003	0.009	0.016		
Historical	0.106	0.065	0.003	0.010	0.055		
Habitat	0.376	0.147	0.314	0.004	0.207		
Other	0.005	0.242	0.003	0.005	0.013		
Background variables						*F* value	*p* value
Sumnep	3.72^A^	3.40^B^	3.69^A^	3.14^C^	3.57	79.50	0.000
						*χ* ^2^	
Country Germany	40.0^A^	49.3^B^	43.7^A^	16.9^C^	39.3	392.87	0.000
Country Finland	49.4^A^	36.5^B^	48.8^A^	37.2^B^	45.1		
Country Latvia	9.8^A^	14.2^B^	7.6^A^	45.9^C^	15.6		
User	67.2^A^	71.0^A^	69.3^A^	83.8^B^	70.6	44.36	0.000
Gender (male)	48.3^AB^	59.0^C^	43.4^B^	54.1^AC^	50.4	28.91	0.000

For continuous variables, in Dunnett’s T3 post hoc test, and for dummy variables, z-test showing significant differences between clusters at the 0.05 level denoted with the letters ^A^, ^B^, ^C^ and ^D^. If Within rows, when values are followed by the same letter there is no significant difference between the clusters

## Discussion and conclusions

This paper presents results of an international study on the relative importance of CES in the Baltic Sea environment for the residents of the three coastal countries: Finland, Germany and Latvia. Relative importance was elicited in a survey question where respondents allocated 100 points between seven categories of CES conforming to the CICES classification of ecosystem services: recreation, landscape, inspiration, learning and education, spiritual experiences and belonging, historically and culturally important places and the existence of habitats. With this compositional measure we avoided the problem often found in importance measures with 5 or 7 point scales, that is, high scores for every item without differences among items.

The results indicate that recreation, habitats and landscapes are considered particularly important CES in the three Baltic Sea countries. Differences in the importance of CES among respondents were explained primarily by the respondents’ country of residence, gender and recreational use of the Baltic Sea. Recreation is the most important CES in all three countries, but habitats are almost as important in Finland and Germany. These country-specific differences are likely related to the sizes, shapes and population densities of the three countries as well as cultural factors. For example, the German Baltic Sea coastline is short compared to its land area and the country stretches further away from the Baltic Sea than the other countries, leading to more emphasis on CES that are less tangible and not associated with the use of coastal and marine areas, including education, spiritual and habitat services. Similar to Van Riper and Kyle (2014), our results reveal that respondents with strong biocentric worldviews emphasize CES related to scientific and biodiversity values of the environment. Respondents who do not use the Baltic Sea for recreation also consider these CES categories more important.

Cluster analyses of CES reveal clear groupings. Interestingly, the existence of habitats forms its own cluster, which implies that people consider the existence of habitats as separate from other CES. Likewise, clustering of respondents indicated that half of them emphasize the importance of habitats over other CES. These two results suggest a distinct status for the existence of habitats among CES for the general public.

Spiritual experiences, inspiration, education and historic and cultural places form a cluster of CES, and are on average seen as less important than the other CES. These notions may help in further developing and operationalizing the ES classification, and indicate how the categories may be combined in future research.

Qualitative and deliberative valuation methods for CES are often suggested to capture the contextuality, subjectivity and plurality of values (see Kenter et al. 2011). These types of methods may inform us about the various dimensions and dynamic characteristics of the CES, but can only engage a limited number of participants. Representativeness can be increased by combining these methods with a quantitative assessment based on surveys among the wider population. Our approach captures the plurality of values, but also provides a quantitative assessment with representative samples of national populations, which are required when the aim is to make comparisons, for example, among the perceptions of various stakeholders or regions.

Lack of data on socioeconomic importance and values precludes quantitative evidence on the entire range of CES in policy assessments. Data on the relative importance of CES can lead to a more encompassing application of the ecosystem-based approach, an important guiding principle in marine policies. The findings in this study can inform policies on the protection and sustainable use of the marine environment. This includes, for example, the EU Biodiversity Strategy, which calls for assessing the state of ecosystem services and their values, and the EU Marine Strategy Framework Directive (2008/56/EC), which requires economic analyses of the marine environment with a focus on ES as one of the recommended approaches. The most important CES (i.e. habitats, recreation and landscapes), can be prioritized in conservation policies while acknowledging that all CES are considered important by some groups of people. The differences in the importance of CES across countries may create challenges for regional policies since countries may place varying emphases on the goals of protection efforts.

Future policy oriented research should focus on the spatial distribution of marine CES and the subsequent benefits from their improved provision. In addition, future research might focus on CES other than recreation, which is the most studied in the Baltic Sea area. This information would also serve the EU Maritime Spatial Planning Directive (2014/89/EU) where assessments of ES can support the planning of maritime activities to ensure sustainable use of the marine environment.

## Electronic supplementary material

Below is the link to the electronic supplementary material.

Supplementary material 1 (PDF 55 kb)
