# Supplementary material for: Cultural ecosystem services provided by the Baltic Sea marine environment
Source: Ambio. 2019 Aug 31;48(11):1350–61. doi: 10.1007/s13280-019-01239-1 (PMC6814646; doi:10.1007/s13280-019-01239-1)
Supplement: Supplementary file 1 — Supplementary material 1 (PDF 55 kb) [file 13280_2019_1239_MOESM1_ESM.pdf]

Ambio

Electronic Supplementary Material
This supplementary material has not been peer reviewed

Title: **Cultural ecosystem services provided by the Baltic Sea marine environment**

Heini Ahtiainen, Eero Liski , Eija Pouta, Katriina Soini, Christine Bertram, Katrin Rehdanz, Kristine Pakalniete, Jürgen Meyerhof

APPENDIX S1

How important do you personally consider the following reasons to value the Baltic Sea and its coastal areas? Please consider your overall motivation to value the Baltic Sea as 100 points and distribute these points among the following reasons according to your motivations. It is not necessary to allocate points to all reasons, so if you do not care about the particular reason, you can write 0. The total must sum to 100 points.

Please distribute points	Points
Opportunities for recreational activities (e.g., swimming, fishing, walking, boating, bird watching)	____
Enjoyment from landscapes	____
Inspiration for artistic work (photographing…)	____
An environment for learning and gaining new information	____
Spiritual experiences, sense of belonging, and symbolic meaning	____
Experiencing historically and culturally important places	____
Habitats for many animals and plants	____
Other reasons not mentioned in the list above	____
Sum	100
